# Influence of the Brain and Nervous System in the Production of Inflammation

**Published:** 1829-01-01

**Authors:** 


### XXIV.

INFLUENCE OF THE BRAIN AND NERVOUS SYSTEM IN THE PRODUCTION OF INFLAMMATION. By Dr. MALDEN, of Worcester.

We have, on many occasions, seized opportunities of opposing the partially fashionable current which certain pathological investigations have lately taken, in respect to the vascular system. The brain and nerves are in danger of being turned out of doors—and we expect soon to find fevers as easily tested by reagents as oxalic or prussic acid! The nervous system is now voted to have nothing to do with the phenomena of inflammation—and when local inflammation leads to constitutional or visceral disorder, it is not at all through the medium of the brain and nerves, but through the instrumentality of "morbid secretions" contaminating the mass of blood, or by extension of the local inflammation along the veins to the viscus affected. In a very sensible paper recently published by Dr. Malden, in our "MIDLAND" cotemporary, a very different and a much more correct view is taken of these things. We are therefore tempted to extract a passage which, in itself, conveys some just notions of disease, and may lead the reader to peruse the original article.

"The more readily sensorial action follows impressions made upon parts, the more readily will Inflammation be produced in them, upon the application of its exciting causes.

"The term pain ought to be employed in Pathology, as a generic term, including all uneasy sensations referred to a part. Nerves must be considered as simply impressible. When a part is stimulated by the application of any irritant, mechanical or chemical, the sensorial action follows, and the effect produced by it is slight or severe, according to the nature of the stimulus, the mode of its application, or the state of the Sensorium.

"It seems probable that the catenated actions which succeed, and which, in their progress, constitute the different grades of Inflammation, are reflex sensorial actions; or the result of a motive impulse, conveyed by the Nerves from the Sensorium, to the part affected, or its vicinity.

"The two extremes of the Arterial system, the Heart and the Capillaries, agree in the following particulars.

"They are both endowed with a contractility which acts independently of the Sensorium, upon the application of stimuli; but they may both be powerfully affected by sensorial action.

"The action of the Heart is modified even in health, by every mental emotion; and anger, shame, and other passions, display, beyond all doubt, as marked an influence on the Capillaries.

"A temporary change then, in the state of the Brain itself, follows the application of a stimulus to a part, which is followed by a reflex motive impulse conveyed by the Nerves. This impulse is not confined to the point upon which the impression has been made, but is communicated to the Arteries surrounding it, increasing the afflux of Blood to them, and by injecting and distending with red globules, minute capillary branches, which before contained only transparent and colourless Serum, either renders them distinctly visible, or, where they are numerous and crowded, produces a diffused redness or blush of greater or less extent.

Thus, simple determination of Blood, as it has been called, is effected by increasing the quantity contained in a certain set of vessels.—' Ubi irritatio ibi fluxus.'

" *Experiment.*—With the point of a needle, gently scratch or prick the back part of the wrist or hand, two or three times, until a slight smarting sensation be produced; in a few seconds a blush will be observed, (not commencing at the stimulated part, and gradually spreading from it) but commencing in several distinct points for some distance round it at the same time, and progressively blending into one blush. These phenomena are easily observed by the naked eye, but, with the assistance of a magnifying glass of slight power, the demonstration is more conclusive and satisfactory.

" This view of the part the Sensorium takes in establishing local determinations in the neighbourhood of a stimulated point, affords the only explanation of the apparently arbitrary manner in which Inflammation is occasionally fixed upon seats to which no cognizable stimulus has been previously applied. The same sensorial change which is produced by the application of a stimulus to any remote part, would, if it should occur independently of the application of such a stimulus, have a tendency to communicate the same motive impulse to the vessels in the neighbourhood of the part, as if a stimulus had been there applied.

" That local determinations can take place from sensorial changes only, is proved from the phenomenon of blushing as the consequence of mental emotion.

" There is a term employed by Medical writers, which, in my opinion, is indicative merely of increased impressibility of the Nervous system—*Mobility.*

" Individuals of delicate structure, or who have been debilitated by dissipation, disease, study, sedentary habits, the puerperal state, or other causes, have this *Mobility*, and are, therefore, hourly subject to some new combination of symptoms, till the Constitution be invigorated, and the Mobility consequently lessened. One of the most marked symptoms attending this state of the habit, is a disposition to irregular flushings; the surface of the body becomes suddenly heated in patches, shifting their seat so rapidly and uncertainly, that, while the Patient is complaining of the flushing in one part, it will leave it and attack another. In this state of the system, the most trifling causes will excite inflammatory action in a part; and if the Mobility, prior to the attack, has been strongly marked, the Physician is seldom called upon to afford his assistance under circumstances more unfavourable; for the readiness with which the Sensorial changes take place, causes an incessant migration of the Inflammatory action from one surface or region to another. Children, after severe attacks of Fever, and Women in the Puerperal state, frequently exhibit this deplorable disposition to shifting Inflammation; and, in these cases, the very depletions we use to combat the Inflammation, by augmenting the Mobility, increase the tendency of the Inflammation to attack different parts in succession. It is upon this principle that, in acute Rheumatism, if large general bleedings be used at the beginning of the disorder, the Inflammatory action shifts more suddenly, and frequently from joint to joint, and even to internal organs, in the progress of the disease, than in those cases where the bleeding has been less profuse."*

* Midland Medical and Surgical Reporter, No. 2. November, 1828.

